# 嵌合抗原受体T细胞治疗成人急性B淋巴细胞白血病中国专家共识（2022年版）

**DOI:** 10.3760/cma.j.issn.0253-2727.2022.02.001

**Published:** 2022-02

**Authors:** 中华医学会血液学分会白血病淋巴瘤学组 中国抗癌协会血液肿瘤专业委员会造血干细胞移植与细胞治疗学组

嵌合抗原受体T细胞（CAR-T细胞）是肿瘤治疗的新兴技术，通过基因工程将针对肿瘤抗原的单链可变区与共刺激分子的基因片段（CAR）整合至T细胞基因组并在T细胞上表达，CAR蛋白的胞外结构特异识别肿瘤抗原，并启动下游信号通路，使CAR-T细胞增殖、活化，发挥靶向肿瘤杀伤效应[1]。2017年8月，美国食品和药物管理局批准全球首个商业化CAR-T细胞产品用于治疗难治/复发急性B淋巴细胞白血病（r/r B-ALL），儿童及年轻成人r/r B-ALL完全缓解（CR）率达85％，1年总体生存率及无病生存率分别达72％及51％[2]。截至2021年11月30日，国内医疗机构共开展209项CAR-T细胞治疗r/r B-ALL的临床研究，其中8项为国家药品监督管理局批准开展的新药临床研究，其余为研究者发起的临床研究。目前CAR-T细胞在r/r B-ALL的常用靶点为CD19和CD22。

鉴于国内已开展多项CAR-T临床研究，并有2项CAR-T产品上市，但临床工作者对CAR-T细胞治疗的指征、细胞制备、疗效评估、并发症诊断与处理等缺乏系统认识。为此中华医学会血液学分会白血病淋巴瘤学组、中国抗癌协会血液肿瘤专业委员会造血干细胞移植与细胞治疗学组组织相关专家编写本项共识，旨在提高临床医护工作者对CAR-T细胞治疗r/r B-ALL的实践能力，为进一步开展临床研究和治疗提供指导意见。

一、入选及排除标准

本共识的入选与排除标准参考目前已上市的CD19 CAR-T细胞治疗ALL临床应用说明[3]，鉴于国内外大部分工作尚处于临床研究阶段，本项共识也汇集了临床研究目前常用的入排标准[4]–[16]。以下入排标准作为参考建议，研究者可根据临床研究的目标进行调整。

（一）入选标准

1. 根据中国抗癌协会血液肿瘤专业委员会、中华医学会血液学分会白血病淋巴瘤学组制定的《中国成人急性淋巴细胞白血病诊断与治疗指南（2021年版）》[17]诊断为r/r B-ALL。难治性白血病：诱导治疗结束（一般指4周方案或Hyper-CVAD方案）未能取得CR/血细胞未完全恢复的CR（CRi）；白血病复发：已取得CR的患者外周血或骨髓又出现原始细胞比例>5％或出现髓外疾病。

2. 白血病细胞经免疫学检测确诊靶抗原阳性。

3. 血清总胆红素、血肌酐≤正常值范围上限2倍，血清ALT和AST≤正常值范围上限3倍。

4. 超声心动图左心室射血分数（LVEF）≥45％。

5. 受试者无肺部活动性感染，非吸氧状态下经皮动脉血氧饱和度≥92％。

6. 乙肝表面抗原阳性患者CAR-T治疗处理参考《靶向B细胞和浆细胞的CAR-T细胞治疗中防治乙型肝炎病毒再激活的中国专家共识（2021年版）》[18]。

7. 预估生存期在3个月以上。

8. ECOG评分0～2分。

（二）排除标准

1. 原发病广泛累及胃肠道、呼吸道、心血管等空腔脏器导致功能障碍的患者。

2. 心电图提示有QT间期延长，既往患有严重心律失常等严重心脏病者。

3. 严重的中枢神经系统（CNS）疾患，如频繁的癫痫发作病史。

4. 伴有严重的活动性感染（单纯性尿路感染、细菌性咽炎除外）。

5. 筛选前4周内有活疫苗接种。

6. 对细胞产品中任何一种成分有过敏史者。

7. 妊娠或哺乳期患者。

二、淋巴细胞采集

目前临床治疗和临床研究主要采用患者自体淋巴细胞制备CAR-T细胞，但在患者因疾病原因无法接受淋巴细胞采集或自体淋巴细胞采集或制备失败时，可考虑采用HLA相合或者半相合供者的淋巴细胞制备异基因来源CAR-T细胞[19]。

患者自体淋巴细胞采集应满足以下要求[20]：采集前12周内无其他异基因细胞治疗史；采集前4周内无聚乙二醇-门冬酰胺酶或供者淋巴细胞输注史；采集前2周内无抗移植物抗宿主病（GVHD）治疗、免疫调节药物、长效G-CSF或长春碱类药物应用史；采集前5 d内无短效G-CSF应用史；采集前3 d内无短效细胞毒药物或系统性糖皮质激素应用史；采集前1 d内行血常规检测，淋巴细胞绝对计数>0.1×10^9^/L（>0.5×10^9^/L为佳）。一般应用血细胞分离机单采单个核细胞，目标细胞量应根据CAR-T细胞制备工艺而定，建议采集1×10^7^以上T淋巴细胞。

对于异基因造血干细胞移植（allo-HSCT）后复发的患者，CAR-T细胞治疗具有潜在的致GVHD风险。对于allo-HSCT后复发患者，大多采用原供者的淋巴细胞进行CAR-T细胞制备，如无法获得原供者淋巴细胞，也可采用受者体内的淋巴细胞进行CAR-T细胞制备。国内外多个临床研究报道allo-HSCT后复发患者接受CAR-T细胞治疗后GVHD发生率不一，供者来源CAR-T细胞治疗后GVHD发生率为4.7％～71.4％[19],[21]–[23]，受者来源CAR-T细胞治疗后GVHD发生率为18.2％～38.5％[22]–[23]；另有研究报道对于半相合移植后复发患者，供者来源CAR-T细胞治疗后GVHD发生风险较受者来源高[23]。为避免CAR-T细胞治疗诱发或加重GVHD，建议allo-HSCT后复发患者在GVHD得到控制的基础上，接受CAR-T细胞治疗，并在CAR-T细胞输注后密切观察GVHD的发生情况。对并发GVHD的患者，临床处理参照《中国异基因造血干细胞移植治疗血液系统疾病专家共识（Ⅲ）——急性移植物抗宿主病（2020年版）》[24]。

三、CAR-T细胞治疗前桥接化疗与预处理方案

对于部分疾病进展迅速的ALL患者需要在淋巴细胞采集术后与CAR-T细胞治疗前进行桥接化疗，桥接化疗方案应充分考虑CAR-T细胞输注时间，一般建议：CAR-T细胞输注前4周内不用聚乙二醇-门冬酰胺酶；CAR-T细胞输注前1周内不用长春碱类、6-巯基嘌呤、6-硫鸟嘌呤、甲氨蝶呤、阿糖胞苷、门冬酰胺酶；CAR-T细胞输注前3 d内不用系统性糖皮质激素、羟基脲、酪氨酸激酶抑制剂[20]。

CAR-T细胞回输前淋巴细胞清除预处理方案：目前国内外常用预处理化疗方案为氟达拉滨25～30 mg·m^−2^·d^−1^×3 d，环磷酰胺250～300 mg·m^−2^·d^−1^×3 d或30 mg·kg^−1^·d^−1^×2 d或500 mg·m^−2^·d^−1^×2 d或750 mg·m^−2^·d^−1^×1 d（可根据患者的情况适当调整），一般于化疗结束第2～3天回输CAR-T细胞[4]–[16]。如患者在接受预处理化疗后合并活动性感染、新发GVHD或预处理化疗相关的严重不良反应（如心肺功能不全、严重低血压等），需暂缓输注CAR-T细胞，待病情控制后再回输，CAR-T细胞输注时间至多推迟至预处理化疗结束后第14天，如超过第14天应根据血象情况再次予以预处理方案化疗[3]。

四、CAR-T细胞输注

患者在输注CAR-T细胞前应置入双腔或三腔中心静脉导管。为预防输注时与二甲基亚砜等低温保存剂有关的输注反应，患者回输低温保存的CAR-T细胞前30～60 min需使用对乙酰氨基酚或苯海拉明等药物[25]。输注CAR-T细胞前，应在患者床边准备吸氧、吸痰等设备和包括肾上腺素在内的紧急抢救药物。告知患者如有气促、皮疹、畏寒、胸痛和背痛等任何不适时，需及时向医护人员报告。CAR-T细胞输注剂量根据不同的产品临床研究推荐剂量，一般为0.5×10^6^/kg～1×10^7^/kg。输注CAR-T细胞时避免使用药液过滤器或白细胞过滤输血器[25]。

输注不良反应包括恶心、呕吐、腹痛、寒战、发热，以及罕见的严重呼吸抑制、神经毒性和心律失常等。发生输注不良反应后处理原则包括减慢或暂停输注CAR-T细胞，再次核查细胞制剂信息。如患者输注不良反应症状不能缓解或持续加重，应立即停止输注，如临床判断上述输注不良反应为过敏导致，应立即进行抗过敏治疗和抢救，并同时检测CAR-T细胞制剂和患者外周血病原微生物，对患者外周血超敏C反应蛋白（CRP）、降钙素原（PCT）、血常规、细胞因子等炎症指标进行检测。如考虑存在病原微生物污染的可能性时，应使用广谱抗生素。有条件的单位在CAR-T细胞回输后，应使用流式细胞术或PCR定期监测体内CAR-T细胞动态变化，为早期判断临床疗效、鉴别诊断CAR-T细胞相关不良反应提供依据。

CAR-T细胞回输后患者可能会发生细胞因子释放综合征（CRS）和其他相关并发症，应定期监测生命体征、24 h出入量、血常规、凝血功能、肝功能、肾功能、电解质、乳酸脱氢酶（LDH）、CRP、铁蛋白、细胞因子（IL-6、IFN-γ等）；建议有条件的单位将CAR-T细胞治疗后出现严重血细胞减少患者转入全环境保护的层流病床接受治疗，严重粒细胞缺乏患者可使用喹诺酮类或含β内酰胺酶抑制剂的复合青霉素制剂和抗真菌药物预防细菌与真菌感染；对既往有癫痫发作病史、白血病中枢神经系统浸润或增强MRI提示脑膜T2信号强化的患者，应考虑进行左乙拉西坦抗癫痫预防性治疗[26]–[27]。

五、CAR-T细胞治疗后并发症评估与处理

（一）CRS分级与处理

CRS是CAR-T细胞输注后最常见的并发症，CAR-T细胞治疗后细胞因子释放导致发热、低血压、低氧血症、心动过速、肝功能损害、肾功能损害、心功能损害、凝血功能障碍等一系列临床症状。结合美国移植与细胞治疗学会（ASBMT/ASTCT）有关CRS的分级标准[27]–[28]，提出以下建议：

1. CRS 1级：表现为发热（体温>38 °C，伴或不伴其他体征），且排除其他发热原因。临床处理：非甾体抗炎药控制体温；排除可能的感染病原（血、尿、痰培养，肺部影像学等）；如合并粒细胞缺乏应用广谱抗生素；如非甾体抗炎药应用后体温大于39 °C超过10 h或持续性发热超过3 d，可考虑应用IL-6受体拮抗剂托珠单抗（tocilizumab），体重30 kg以下每次应用12 mg/kg，体重30 kg及以上每次应用8 mg/kg，单次剂量不超过800 mg，每8 h可重复给药，24 h内给药不超过3次。

2. CRS 2级：表现为发热伴低血压（不需应用升压药）和（或）低氧血症（需要低流量吸氧）。临床处理：在上述1级CRS临床处理方案基础上，应用生理盐水10～20 ml/kg加强补液治疗，如有必要可重复补液以维持血压；如低血压经补液治疗效果不佳，可考虑应用托珠单抗；低流量吸氧支持；如患者具有重度CRS高危因素（CAR-T细胞输注3 d内出现CRS表现、高肿瘤负荷、合并其他基础疾病）或低血压经托珠单抗治疗效果不佳或低灌注症状进展迅速，应用地塞米松10 mg每6 h 1次或甲泼尼龙1～2 mg/kg每6～12 h 1次。

3. CRS 3级：表现为发热伴低血压（需要一种血管活性药维持血压）和（或）低氧血症（需要高流量鼻导管、面罩吸氧，无需机械通气）。临床处理：在2级CRS临床处理方案基础上，应用血管活性药物维持血压并进行心脏超声评估；应用托珠单抗联合糖皮质激素治疗，如疗效不佳地塞米松可加量至20 mg每6 h 1次；应用高流量吸氧。如果上述治疗效果不佳，可考虑行血浆置换[29]。

4. CRS 4级：表现为发热伴低血压（需要多种升压药，但不包括血管加压素）和（或）低氧血症（需正压机械通气，包括CPAP、BiPAP和气管插管）。临床处理：患者应转移至ICU加强监护治疗；继续应用补液、托珠单抗、糖皮质激素、血管活性药物治疗，加强血流动力学监测；可应用大剂量甲泼尼龙（1 g/d）冲击治疗，如临床症状好转，迅速减量；应用正压机械通气维持呼吸功能。如果上述治疗效果不佳，可考虑行血浆置换[29]。

（二）免疫效应细胞相关神经毒性综合征（ICANS）分级与处理

ICANS是CAR-T细胞等免疫效应细胞引起的神经系统毒性，表现为神经及精神系统一系列临床症状，包括精神状态的改变、失语、不同程度的意识障碍、偏瘫或癫痫等[28]。ICANS的诊断主要根据免疫效应细胞相关脑病（ICE）评分（患者定向力、命名、指令执行、书写、计数能力），结合患者临床症状和影像学改变，共分4级。根据ASTCT有关ICANS的分级标准[27]–[28]，提出以下建议，此建议主要适用于成人。

1. ICE评分：由医务人员在床边对患者能力进行测定，满分10分：①定向力（4分）：患者准确描述当前年、月，所在城市、医院等4项；②命名（3分）：命名三个对象，如时钟、笔、钮扣等；③指令执行（1分）：可执行简单指令（如展示2根手指、闭眼、伸舌等）；④书写（1分）：写一个标准句子；⑤计数能力（1分）：从100开始以10为单位倒数。

2. ICANS 1级：ICE 7～9分，患者可自主苏醒。临床处理：防止误吸，吸氧补液，暂禁食、禁饮；评估吞咽功能，若吞咽能力受损，将所有口服药物和（或）营养物质转换为静脉注射；避免使用抑制中枢神经系统的药物；对于烦躁不安的患者，可以使用低剂量的劳拉西泮或氟哌啶醇；眼底镜检查以评估视乳头水肿程度；头颅平扫/增强MRI；诊断性腰椎穿刺，测量脑脊液压力；如患者有局灶性周围神经功能缺损，则行相关椎体MRI；若无法行MRI检查，可选择CT；如果ICANS同时并发CRS，则考虑使用托珠单抗8 mg/kg静脉滴注进行抗IL-6治疗；如有持续癫痫状态请神经内科协同治疗。

3. ICANS 2级：ICE 3～6分，患者可通过声音唤醒。临床处理：在1级ICANS处理方案基础上，可静脉使用地塞米松10 mg每6 h 1次，或静脉使用甲泼尼龙1 mg/kg每12 h 1次；若伴有≥2级的CRS，则考虑转入ICU治疗。

4. ICANS 3级：ICE 0～2分，患者可通过疼痛刺激唤醒或癫痫发作经临床治疗可获得控制或神经影像学表现为局灶性脑水肿。临床处理：在2级ICANS处理方案基础上，建议患者转移至ICU；神经内外科会诊协同诊治；如患者ICANS持续≥3级，则考虑每2～3 d重复神经影像学（MRI或CT）检查。如患者伴有3级及以上视乳头水肿，伴影像学脑水肿征象，或脑脊液压力≥20 mmHg（272 mmH_2_O），需进行脑水肿对症处理：应用大剂量糖皮质激素如地塞米松10 mg每6 h 1次或甲泼尼龙1 g/d；将患者床头端抬高至30度；应用甘露醇或高渗盐水进行脱水治疗；如果患者装有Ommaya囊，引流脑脊液至脑脊液压力<20 mmHg（272 mmH_2_O）；每日行头颅CT，并根据临床情况调整用药，以防止脑水肿复发。

5. ICANS 4级：ICE 0分；患者不能唤醒或需要反复的疼痛刺激唤醒；危及生命的持续性癫痫发作；严重运动功能障碍，如偏瘫或瘫痪；神经影像学上弥漫性脑水肿。临床处理：在3级ICANS处理方案基础上，可考虑机械通气；如患者出现惊厥性癫痫持续状态，在神经内科医师指导下控制癫痫发作，可应用苯巴比妥、劳拉西泮等治疗，持续脑电图监测。

（三）血细胞减少与感染

B-ALL患者接受靶向CD19 CAR-T细胞治疗后3～4级中性粒细胞减少发生率为53％～94.3％，3～4级贫血发生率为51.4％～68.0％，3～4级血小板减少发生率为41％～53％，其中3～4级中性粒细胞减少持续时间可长达14～19.5 d[4],[30]–[32]。

1. 预防性措施：接受CAR-T细胞治疗的患者应做到全环境保护，有条件者建议在无菌层流设施中接受CAR-T细胞治疗；注意保持口腔、消化道、生殖道清洁；同时避免剧烈运动。

2. 血制品输注：患者贫血症状明显、血红蛋白<60 g/L应及时输注红细胞。对血红蛋白≥60 g/L而体能状况较弱、耐受性较差的患者也应根据临床情况及时输血。当血小板计数<20×10^9^/L或有出血症状可输注辐照血小板，当血小板输注无效时应输注HLA配型血小板。合并有凝血功能异常时应及时输注凝血酶原复合物、新鲜冰冻血浆、纤维蛋白原或冷沉淀改善凝血功能。

3. 集落刺激因子：因考虑髓系集落刺激因子可能与CRS发生有关，CAR-T细胞回输后两周内或CRS症状缓解前慎用髓系集落刺激因子，避免应用粒细胞-巨噬细胞集落刺激因子[3],[33]。

4. 粒细胞缺乏伴发热：对粒细胞缺乏伴发热患者应使用经验性抗生素治疗，并积极进行微生物学和影像学检查，明确病原微生物和感染部位，根据病原微生物及药敏结果调整抗生素方案；应至少每3 d复查1次全血细胞计数、肝肾功能、电解质、PCT和CRP；具体参考《中国中性粒细胞缺乏伴发热患者抗菌药物临床应用指南（2020年版）》进行治疗[34]。

（四）B细胞缺陷

由于CAR-T细胞靶向的CD19、CD22等抗原为B细胞特异且广泛表达，CAR-T细胞靶向杀伤B-ALL细胞同时，还会清除表达CD19、CD22等的正常B细胞，从而导致B细胞免疫功能缺陷。建议CAR-T细胞治疗后应定期复查B淋巴细胞数量和免疫球蛋白。每月至少1次静脉注射丙种球蛋白10 g，并将IgG维持在4 mg/L以上。建议患者CAR-T细胞治疗后连续3个月应用甲氧苄氨嘧啶-磺胺甲唑（如果过敏可选择喷他脒）和阿昔洛韦/伐昔洛韦分别预防肺孢子菌和单纯疱疹病毒/水痘带状疱疹病毒[20]。

目前关于CAR-T治疗后如何进行免疫接种尚缺乏足够证据[20],[27],[35]。欧洲血液与骨髓移植协会（EBMT）和ASTCT联合发布的专家共识建议在患者接受CAR-T细胞治疗后至少6个月再行预防接种。应优先给患者接种灭活流感疫苗、13-价肺炎链球菌疫苗和流感嗜血杆菌疫苗[20]。

（五）噬血细胞性淋巴组织细胞增生症/巨噬细胞活化综合征（HLH/MAS）

HLH/MAS是一组由于促炎性细胞因子大量释放、巨噬细胞和淋巴细胞的过度活化、伴随吞噬血细胞现象的综合征。在CAR-T细胞治疗过程中，HLH/MAS常继发于中重度CRS，有研究者报道其发生率小于1％[27]。临床表现为持续性发热、肝脾大、全血细胞减少，以及骨髓、肝、脾、淋巴组织发现噬血现象等。

传统HLH/MAS诊断标准不完全适合CAR-T治疗相关的HLH/MAS，国际上部分学者提出CAR-T细胞治疗相关HLH/MAS往往有以下临床表现：铁蛋白水平>10 000 µg/L、肝功能不全（胆红素、转氨酶升高）、肾功能不全（尿量减少、血肌酐升高）、呼吸功能不全（影像学有肺水肿证据）以及骨髓穿刺/组织器官活检提示组织细胞噬血现象[27]。

CAR-T细胞治疗相关HLH/MAS一线治疗方案可按3级CRS处理方案，应用托珠单抗联合糖皮质激素治疗，根据病情，可考虑加用芦可替尼5～10 mg每日2次治疗[36]，或行血浆置换，每次3 000 ml，连续3 d[29]。初始治疗48 h后无改善者可加用依托泊苷75～100 mg/m^2^，根据临床表现和血清学检查，4～7 d后可重复使用。

六、复发的预防与治疗

r/r B-ALL患者经CAR-T细胞治疗后可取得近90％的CR率，但接近半数患者仍将复发。有多种因素与CAR-T细胞治疗后复发有关：①患者CAR-T治疗前疾病状态：CAR-T治疗前高肿瘤负荷、中枢神经系统白血病累及、髓外疾病、CAR-T治疗后MRD持续阳性或转阳、经CAR-T治疗无效或复发、allo-HSCT治疗后复发、高危细胞遗传学及分子生物学异常（如TP53突变、KMT2A基因重排）。②CAR-T细胞制备质量与治疗过程：CAR-T细胞制备转染效率低、体外增殖能力差、应用异基因CAR-T细胞产品、CAR-T治疗后患者体内CAR-T细胞扩增低下、CAR-T细胞耗竭标志表达升高[37]。

目前预防复发有以下策略：①CAR-T治疗后严密监测MRD和B淋巴细胞，如MRD转阳或持续升高，对有靶向药物靶标的患者进行靶向治疗，或进行联合化疗。②CAR-T细胞治疗桥接allo-HSCT，CAR-T细胞治疗后是否需要桥接allo-HSCT目前国际上尚无定论，国内多家医疗机构临床研究表明，CAR-T细胞治疗桥接allo-HSCT可以显著减少CAR-T治疗后复发，提高患者长期生存率。并且研究表明在CAR-T治疗后MRD阴性阶段桥接allo-HSCT更加有助于减少复发，CAR-T细胞治疗不增加allo-HSCT相关并发症[38]–[40]；另有国际专家共识建议对具有上述复发高危因素患者在CAR-T治疗后3～6个月桥接allo-HSCT[37]。③国内多家医疗机构临床研究表明，不同靶点CAR-T细胞序贯治疗，如靶向CD19 CAR-T细胞序贯靶向CD22 CAR-T细胞治疗显著降低患者复发率，提高患者无病生存率[11],[41]。

七、CAR-T细胞治疗疗效评估与随访

CAR-T治疗后应根据《中国成人急性淋巴细胞白血病诊断与治疗指南（2021年版）》[17]对患者骨髓、中枢神经系统、髓外疾病进行原发病疗效评估。

目前尚无前瞻性研究提供CAR-T治疗后随访方案，建议：CAR-T治疗后前半年每1～2个月、半年后每3～6个月复查骨髓，其中骨髓MRD监测除了通过常规的流式细胞术，可通过更加敏感手段评估，如融合基因或特定基因突变的实时定量PCR、Ig高通量测序等[42]。每月检测1次外周血B细胞（流式细胞术）、免疫球蛋白水平、全血细胞计数、肝肾功能和电解质。
